# β-catenin regulates HIV latency and modulates HIV reactivation

**DOI:** 10.1371/journal.ppat.1010354

**Published:** 2022-03-07

**Authors:** Hannah J. Barbian, Melanie S. Seaton, Srinivas D. Narasipura, Jennillee Wallace, Reshma Rajan, Beverly E. Sha, Lena Al-Harthi

**Affiliations:** 1 Department of Microbial Pathogens and Immunity, Rush University Medical Center, Chicago, Illinois, United States of America; 2 Department of Internal Medicine, Division of Infectious Diseases, Rush University Medical Center, Chicago, Illinios United States of America; Institut Cochin, INSERM U1016, FRANCE

## Abstract

Latency is the main obstacle towards an HIV cure, with cure strategies aiming to either elicit or prevent viral reactivation. While these strategies have shown promise, they have only succeeded in modulating latency in a fraction of the latent HIV reservoir, suggesting that the mechanisms controlling HIV latency are not completely understood, and that comprehensive latency modulation will require targeting of multiple latency maintenance pathways. We show here that the transcriptional co-activator and the central mediator of canonical Wnt signaling, β-catenin, inhibits HIV transcription in CD4+ T cells via TCF-4 LTR binding sites. Further, we show that inhibiting the β-catenin pathway reactivates HIV in a primary T_CM_ cell model of HIV latency, primary cells from cART-controlled HIV donors, and in CD4+ latent cell lines. β-catenin inhibition or activation also enhanced or inhibited the activity of several classes of HIV latency reversing agents, respectively, in these models, with significant synergy of β-catenin and each LRA class tested. In sum, we identify β-catenin as a novel regulator of HIV latency *in vitro* and *ex vivo*, adding new therapeutic targets that may be combined for comprehensive HIV latency modulation in HIV cure efforts.

## Author summary

There is no cure for HIV despite over 30 years of research. An HIV cure will rely on understanding cellular pathways that regulate HIV latency, which can be exploited for cure strategies. We report here for the first time that transcriptional co-activator β-catenin regulates HIV latency in the primary target for HIV latency, the CD4+ T cell population. We also show that its manipulation can reactive or repress HIV alone or in combination with known modulators of HIV latency. Together, our findings classify β-catenin as a regulator of HIV latency with significant applications to cure strategies.

## Introduction

Despite progress in treating HIV infection and extending the lifespan of HIV-infected individuals, HIV remains an incurable disease, with patients requiring continuous combination antiretroviral therapy (cART). Cessation of cART results in a rebound of HIV viremia from latently infected cells, which persist throughout the lifetime of the infected individual. This latent reservoir is the main barrier to an HIV cure and is thus the primary target of HIV cure strategies.

The latent reservoir is thought to be seeded within days of infection [1] via direct infection of CD4+ T cells in a resting state, or from infection of activated CD4+ T cells which later transition to a quiescent state [2]. Additionally, other long-lived cell types or cells located in anatomical sites with suboptimal diffusion of cART such as lymphoid tissues or the central nervous system may contribute to the persistent viral reservoir [3–7]. In latently infected cells, replication-competent integrated HIV provirus remain in a transcriptionally suppressed state. Several mechanisms mediating the establishment and maintenance of this transcriptional silencing have been defined. The propensity of HIV to integrate into actively expressed genes may result in transcriptional interference, whereby RNA polymerase “read-through” of upstream host genes results in displacement of transcription factors from the HIV LTR [8, 9]. Binding of transcription factors such as NF-κB, C/EBP, Sp-1, and AP-1 are crucial for active HIV transcription. Access to transcription factors is limited by the quiescent state of latent reservoir CD4+ T cells, which have characteristically low levels of transcriptional activators. For example, key HIV transcription factor NF-κB is sequestered in the cellular cytoplasm by inhibitors of NF-κB in resting CD4 T cells. Access to HIV elongation factor P-TEFb is also critical for processive HIV transcription, and is similarly decreased during CD4 T cell quiescence and in latently infected cells [10]. Chromatin modifications and nucleosome binding of the HIV provirus can further occlude access of transcription and elongation factors to the HIV LTR [11]. Thus, the coordination of HIV transcriptional silencing is multi-factorial, with numerous host factors contributing to the establishment and maintenance of HIV latency. However, the cellular pathways involved in HIV latency are likely far from fully understood, indeed, recent shRNA screen and single-cell analysis have revealed several potentially novel modulators of HIV latency [12, 13].

The pathways involved in HIV latency have been targeted in strategies for either a sterilizing or functional HIV cure, although new cure strategies aimed at eliminating integrated HIV genomes in the latent reservoir using gene editing approaches are also evolving [14, 15]. Sterilizing HIV cure strategies aim to eradicate all cells containing replication competent provirus by reversing transcriptional silencing and inducing viral transcript and protein production that make the cell vulnerable to death through cytopathic effects of the infected cell, circulating immune cells, or by immunotherapies. This method, termed “shock and kill,” largely relies on small molecule drugs which target the cellular pathways governing HIV latency. For example, drugs targeting protein kinase C (PKC), including prostatin and bryostatin-1, lead to the activation of NF-κB and other transcription factors; HDAC inhibitors like SAHA release HIV from chromatin; and BET inhibitors like JQ1 promote recruitment of P-TEFb to the HIV LTR [16]. On the other hand, functional cure strategies aim to permanently silence HIV to produce a state of deep latency during which cART therapy can be stopped without viral reactivation. This method, termed “block and lock”, targets the same cellular pathways but towards the opposite effect, such as by inhibiting Tat transactivation [17]. Thus, understanding the pathways controlling HIV latency and using small drugs to manipulate this latency control may lead to a sterilizing or functional HIV cure.

Clinical trials testing latency reversing agents (LRAs) in HIV+ patients showed transient increases in HIV transcripts and some increase in plasma viremia, however, no study to date has resulted in a significant decrease in the HIV reservoir [18–22]. More recently, even “second generation” LRAs, which show promise in potent latency reversal without global T cell activation, fail to show decreases in the overall size of the viral reservoir in animal models [23, 24]. Failure of potent LRAs to effectively reduce the HIV reservoir may be due to incomplete performance in heterogeneous patients, cell or tissue types, with some cells remaining refractory to latency reversal [25–28]. To address this issue, combinations of synergistic LRAs and repeated doses may vastly improve the efficacy of “shock and kill” strategies [29]. Indeed, combination treatment with different classes of LRAs have shown promising improvements in overall LRA activity [30, 31]. Effective latency reversal will thus likely require a combination of LRAs targeting multiple pathways involved in HIV latency maintenance to ensure comprehensive activity in the diverse cell and tissue types that comprise the latent cell reservoir.

Pathways controlling HIV transcription often play a role in the establishment or maintenance of HIV latency. β-catenin is a transcriptional coregulator and a central mediator of the canonical Wnt/β-catenin pathway, which is involved in the transcriptional activity of myriad genes impacting cell proliferation, differentiation, apoptosis, and communication [32]. Recently, we showed that Wnt/β-catenin signaling is responsible for the non-cytolytic ability of CD8+ T cells to suppress HIV transcription in CD4+ T cells, begging the question of whether it could also account for the recently appreciated role of CD8+ T cells to maintain HIV latency in CD4+ T cells [24, 33, 34]. Here, we investigate the role of β-catenin in modulation of HIV transcription and latency in CD4+ T cells. We find that β-catenin acts upon the HIV LTR to regulate HIV transcription in CD4+ T cells and can be used to modulate HIV latency either by promoting or inhibiting reactivation *in vitro* and *ex vivo*. These results define β-catenin as a novel pathway by which HIV latency is maintained in the latent reservoir. Our findings also place the β-catenin pathway at the interface of shock and kill and block and lock cure strategies, as latency can be modulated by inhibiting the pathway to activate latent HIV or inhibiting the pathway to activate HIV.

## Results

### β-catenin modulates HIV transcription in primary CD4+ T cells at the LTR

**Fig 1:**
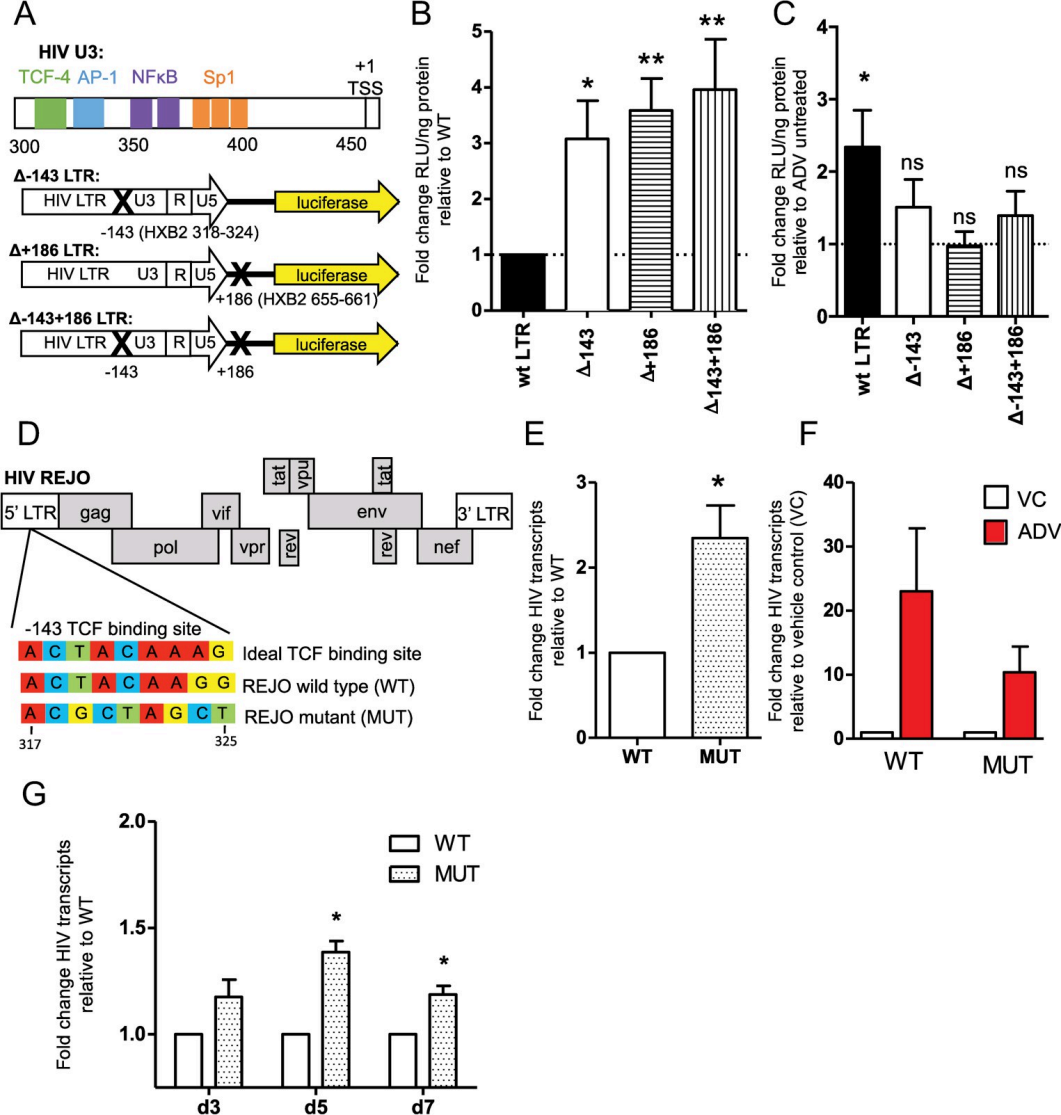
β-catenin modulates HIV transcription in CD4+ T cells via TCF-4 binding at the LTR. (A) Schematic showing the -143 TCF-4 binding site relative to other transcription factor binding sites on the HIV LTR and with HXB2 numbering, and the LTR luciferase reporter plasmid constructs, containing deleted TCF binding sites shown with X marks at position -143, +186, or both -143 and +186 relative the wildtype HIV BaL transcription start site. (B) CD4+ T cells isolated from n = 5 healthy donors were activated for 24 hrs then nucleofected with LTR reporter constructs. LTR activity was quantified using luciferase relative light units normalized to total protein concentration of the cell lysate. Fold change in LTR activity of the TCF binding site mutants is shown relative to the wildtype HIV BaL LTR control. Nucleofections were performed in duplicate for each donor. (C) CD4+ T cells were nucleofected with LTR reporter constructs as above but were treated with 200 nM adavinint (ADV) 2 hours post-nucleofection. Fold change in LTR activity of wildtype and mutant TCF-4 binding sites are shown relative to cells not treated with ADV. (D) Schematic showing the HXB2 position and sequence of wildtype HIV-REJO TCF binding site compared to the ideal TCF binding site sequence, as well as the mutated TCF binding site in the U3 region of the 5’ LTR of REJO full length molecular clone. (E) CD4+ T cells isolated from n = 6 healthy donors were stimulated for 24 hours then infected with wildtype (WT) or TCF binding site mutant (MUT) HIV REJO via spinoculation with 0.01 MOI virus. Cells were harvested at 24 hours and intracellular HIV transcripts were quantified relative to GAPDH. Fold increase of MUT over WT virus is shown. (F) CD4+ T cells isolated from 4 healthy donors were infected after 24 hours stimulation. 100nM adavivint (ADV) was added following spinoculation, cells were harvested at 24 hours and HIV transcripts quantified as above. Fold change relative of ADV treated cells (red) to DMSO control for wildtype and mutant virus is shown. (G) PBMCs were isolated from 3 healthy donors, stimulated for 3 days, then spinoculated with 0.01 MOI wildtype and TCF binding site mutant virus. Infected cells were harvested at 3, 5, and 7 days post infection, intracellular HIV transcripts were quantified as above. Fold change of TCF binding site mutant to wildtype infected cells harvested on the same day are shown. Columns indicate mean with SEM error bars for all panels. Significance was determined using paired *t*-tests for all panels, * p<0.05, ** p<0.01.

To determine whether β-catenin acts on the HIV LTR via its effector TCF-4 to inhibit HIV transcription in CD4+ T cells, we generated LTR reporter plasmids containing 7 or 8-nucleotide deletions in predicted TCF-4 binding sites at positions -143 and +186 relative to the transcriptional start site (corresponding to HXB2 positions 318–324 and 655–661, respectively), as well as a double mutant lacking both TCF-4 binding sites (Fig 1A). Both sites were previously identified to bind TCF-4 in complex with β-catenin by chromatin immunoprecipitation [35]. We found that deletion of one or both TCF-4 binding sites led to 3.1- to 4.0-fold increase in LTR activity compared to wildtype control in activated CD4+ T cells isolated from healthy donors (n = 5 donors, p = 0.014, 0.001, and 0.009 for Δ-143, Δ+186, and Δ-143+186 mutants, respectively) (Fig 1B). Inhibition of the Wnt/β-catenin pathway by the small molecule inhibitor adavivint (SM04690, ADV), which inhibits canonical signaling and decreases levels of β-catenin [36], increased the activity of wildtype LTR reporter by 2.3-fold (p = 0.028), whereas it had no significant effects on the LTR reporters containing TCF-4 binding site mutations (Fig 1D). These data demonstrate that β-catenin inhibit HIV transcription in activated CD4+ T cells via the LTR.

We next assessed the effect of β-catenin on inhibition of HIV transcription of a transmitted/founder virus (HIV REJO). We synthesized a mutated -143 TCF-4 binding site and used Gibson assembly to insert the mutated site in the 5’ LTR of pREJO.c full-length infectious molecular clone [37] (Fig 1D). REJO contains a 1-nucleotide mutation relative to the ideal TCF-4 binding site sequence, which is ACWWCAAAG (Fig 1D). We previously found that up to two TCF-4 binding site mutations still allow TCF-4 binding at the HIV LTR, albeit with less affinity compared to more intact sites [35]. Analysis of 1,364 sequences from the Los Alamos National Laboratory HIV sequence database finds that 89% of HIV-1 subtype B sequences have a TCF-4 binding site within two mutations of the ideal binding sequence, indicating that this site is fairly well conserved amongst HIV strains. Following a single infection cycle in activated CD4+ T cells isolated from healthy donors (n = 6), we found that the TCF-4 binding site mutant virus showed a 2.3-fold increase in HIV transcripts compared to wildtype HIV-REJO (p = 0.016) (Fig 1E). Inhibition of the β-catenin pathway via treatment of CD4+ T cells with adavivint at the time of infection resulted in a 23-fold increase in HIV transcripts in wildtype HIV-REJO whereas the mutant virus showed 10-fold increase in HIV transcripts, although the difference between strains was not significant (Fig 1F). Residual effect of β-catenin inhibition in the -143 TCF-4 binding site mutant could be due to alternate intact TCF-4 binding sites in the LTR, such as at position +186, or LTR-independent effects of β-catenin. Infection of activated whole PBMCs (n = 3 donors) over 7 days showed a modest yet significantly increased HIV transcripts in the TCF-4 binding site mutant infected cells, with 1.4 to 1.2 fold increase in intracellular HIV RNA on day 5 and day 7 post-infection (p = 0.018 and 0.043, respectively) (Fig 1G). More modest increases in HIV transcription over multiple rounds of replication may be due to waning active β-catenin levels over time, which are increased in CD4+ T cells by activation [38]. Together, this suggests that β-catenin is a robust inhibitor of HIV transcription in CD4+ T cells, acting specifically at the HIV LTR via binding partner TCF.

### Inhibition of the β-catenin pathway reactivates HIV from latency

**Fig 2:**
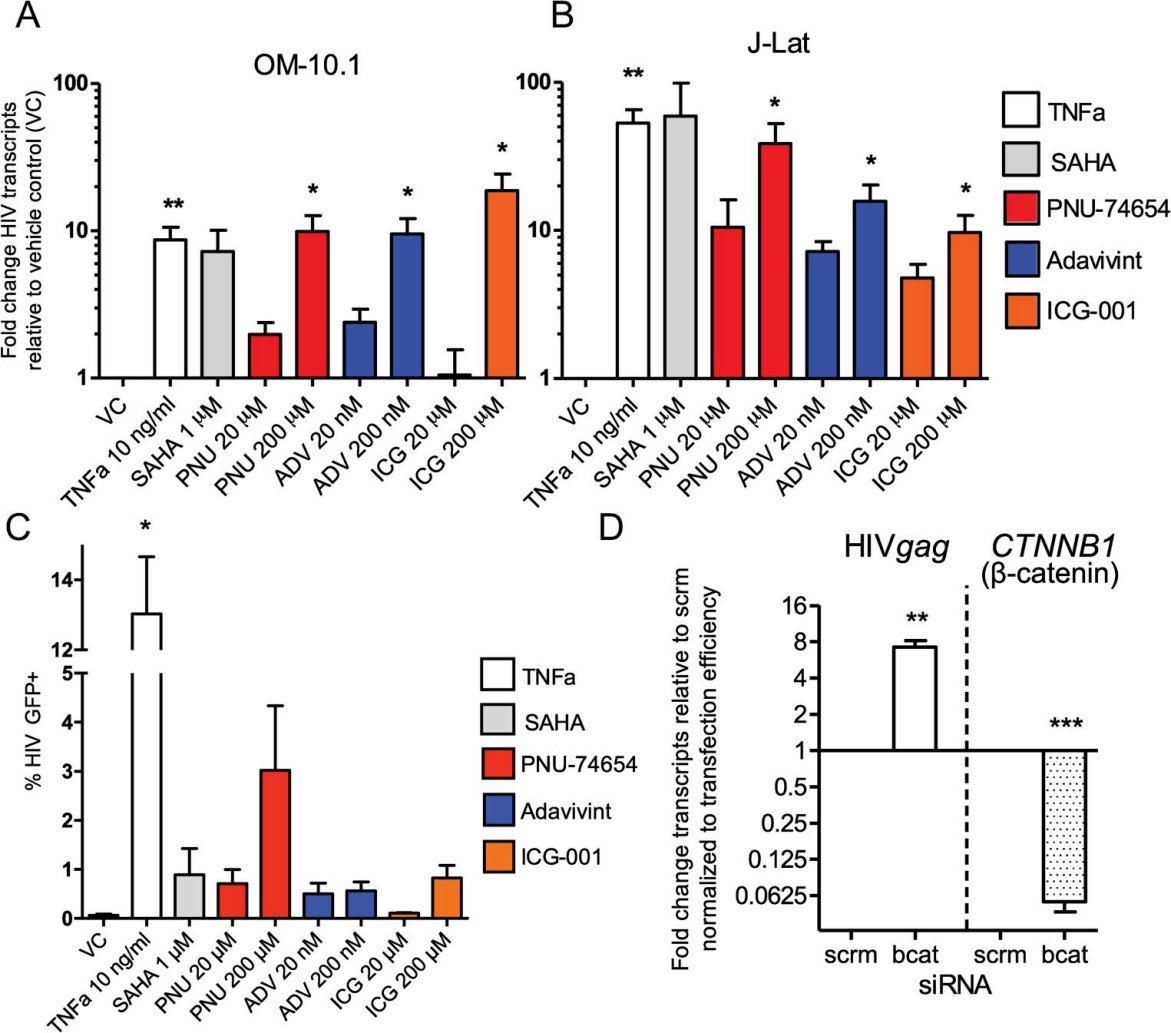
Inhibition of the β-catenin pathway reactivates HIV in cell lines. Treatment of OM-10.1 (A) and J-Lat (B) cells with known latency reversing agents TNFα, SAHA, or β-catenin inhibitors PNU-74654 (red), adavivint (blue), and ICG-001 (orange) for 48 hours. Two concentrations of β-catenin inhibitors were tested. Fold change of cellular HIV transcripts relative to DMSO vehicle control is shown, treated conditions were compared to controls from the same experiment. (C) Percent of HIV GFP+ cells following drug treatments are shown. (D) siRNA knockdown of β-catenin in J-Lat cells was performed for 24 hours. Fold change of HIV gag (left) and CTNNB1 (the gene encoding β-catenin, bcat, right) intracellular transcripts to scrambled control siRNA (scrm) is shown (left). Values were normalized to nucleofection efficiency (S1H Fig). Columns indicate mean of 6 (A and B) or 3 (C) replicates with SEM error bars for. Significance was determined using paired *t*-tests for all panels, * p<0.05, ** p<0.01, *** p<0.001.

The ability of β-catenin to inhibit HIV transcription suggests a possible role in maintaining HIV latency. To test whether β-catenin can control HIV latency, we utilized latent T cell clones OM-10.1 and J-Lat 8.4, which contain an integrated HIV genome yet maintain very low levels of HIV transcription and protein production [39, 40]. We tested ADV and two additional inhibitors of β-catenin pathway activity, PNU-74654 (PNU, inhibits the interaction between β-catenin and TCF), and ICG-001 (ICG, which binds to the transcriptional coactivator CBP to interfere with CBP interaction with β-catenin to inhibit the pathway). All three inhibitors significantly decreased β-catenin signaling through TCF-4 binding sites (S1A Fig), ADV decreased protein levels of β-catenin at concentrations as low as 20 nM (S1B and S1C Fig), and ADV and ICG decreased protein levels of downstream target c-Myc (S1B and S1D Fig) over 5-fold in J-Lat cells at 200 nM and 200 μM, respectively, demonstrating anti-β-catenin activity in these cells. We found that all three small molecule inhibitors of Wnt/β-catenin potently induced HIV transcription in latent cell lines (Fig 2A and 2B). In OM-10.1 cells, PNU (200 μM), ICG (200 μM), and more potent inhibitor ADV (200 nM) induced HIV RNA levels 10-, 19-, and 10-fold over vehicle control, respectively, on par with known potent HIV latency reversing drugs TNFα (10 ng/ml) and SAHA (1 μM), which induced 9- and 7-fold increase in HIV transcripts (Fig 2A), albeit at much higher concentrations for PNU and ICG, which were associated with some cellular toxicity (S1E Fig). In J-Lat cells, these inhibitors elicited a 10- to 39-fold increase in HIV transcripts, approaching the level of positive controls TNFα and SAHA, which induced 53 to 59-fold increase in HIV transcription (Fig 2B) with little loss of cell viability (S1F Fig). These inhibitors resulted in modest reactivation of HIV proteins, as quantified by detection of intracellular HIV GFP reporter, with a maximal reactivation of 3.0, 0.6, and 0.8% of cells for PNU, ADV, and ICG, respectively, which was similar to that of SAHA (0.9%), but significantly less than TNFα (13.0%) (Figs 2C and S1G). To test the specificity of β-catenin in the latency reversing potential of these drugs, we knocked down β-catenin levels in J-Lat cells using siRNA and showed that HIV transcription increased 7-fold following knockdown (Figs 2C and S1H). This data suggests that β-catenin is involved in the maintenance of HIV latency, with its inhibition resulting in robust initiation of transcription in latently infected cells.

### β-catenin pathway inhibition reactivates HIV from latency in an *ex vivo* primary cell model

**Fig 3:**
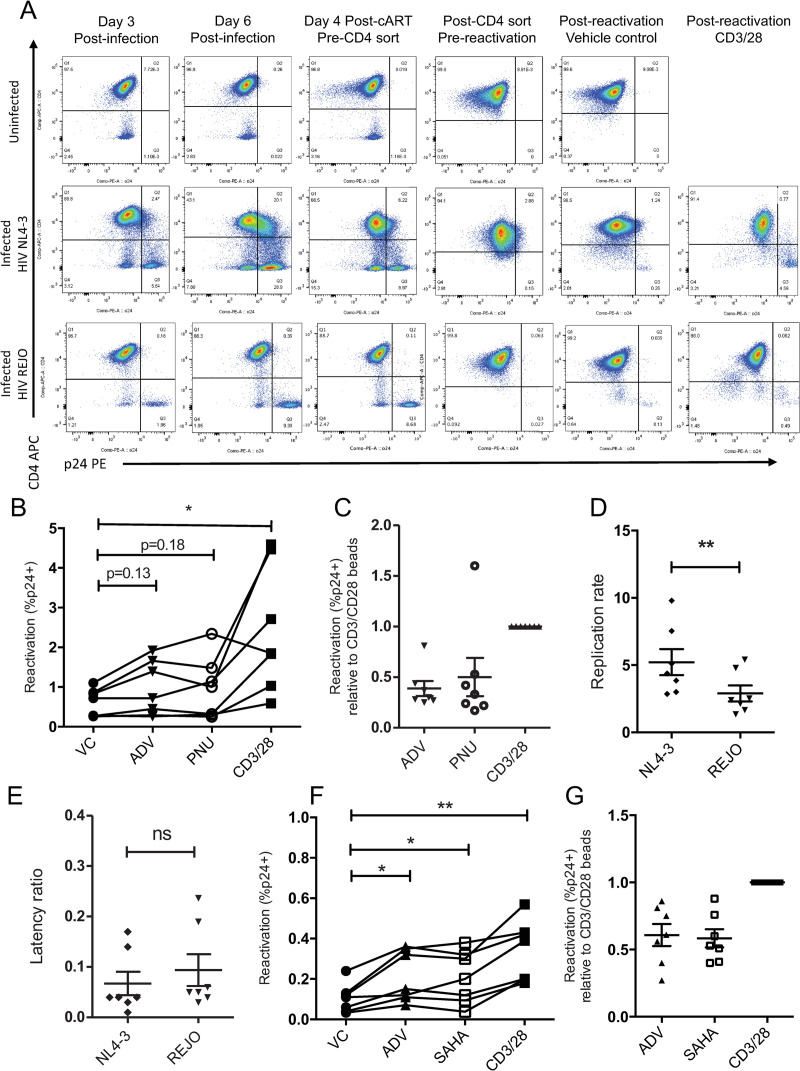
β-catenin pathway inhibition reactivates two HIV strains in a primary T_CM_ model of HIV latency. (A) Surface CD4 and intracellular HIV p24 were measured by flow cytometry throughout the establishment of latently infected T_CM_ cells and following reactivation. Uninfected control (top row), HIV NL4-3 infected (middle row), and HIV REJO infected (bottom row) cultures from one representative donor are shown. (B) Reactivation of latent HIV NL4-3, measured as the percentage of CD4 negative HIV p24 positive cells, in 7 donors treated with β-catenin inhibitors adavivint (ADV, 100 nM), PNU-74654 (100 μM), and positive control αCD3/αCD28 activation beads is shown with lines connecting cells from the same donor. (C) Reactivation of latent NL4-3 with ADV and PNU relative to the % reactivation with αCD3/αCD28 activation beads. (D) The replication rate, measured as the percentage of CD4 negative HIV p24 positive cells at day 6 post-infection over day 3 post-infection, in 7 donors infected with viruses HIV NL4-3 and REJO are shown. (E) The latency ratio, quantified as the percentage of CD4 negative HIV p24 positive cells following reactivation with positive control CD3/28 beads over the percentage of CD4 negative HIV p24 positive cells at peak infection, for 7 donors infected with viruses HIV NL4-3 and REJO are shown. (F) Reactivation of latent HIV REJO, quantified as the percentage of CD4 negative HIV p24 positive cells, for 7 donors treated with β-catenin inhibitor ADV (100 nM) and known latency reversing agents SAHA (1 μM) and CD3/CD28 beads is shown with lines connecting each donor. (G) Reactivation of latent REJO with ADV and SAHA relative to the % reactivation with αCD3/αCD28 activation beads. Statistical p-values were determined via repeated measures ANOVA with Dunnett’s multiple comparisons test for panels B and F, while paired *t*-test were used for panels c, d, e, and g; * p<0.05, ** p<0.01, *** p<0.001.

To determine the effect that β-catenin pathway inhibition has in primary human cells, we utilized a cultured central memory T cell (T_CM_) model of HIV latency [41]. Primary latency models have the advantage over latent T cell lines of including HIV integration site and donor variation, as well as modeling the dynamic active/quiescent states of primary T cells. This T_CM_ model utilizes primary naïve CD4+ T cells that have been skewed to a quiescent T_CM_ phenotype and are infectible with replication competent, full-length HIV NL4-3, and has been extensively validated by others [41–44]. Following infection of T_CM_ cells, latency is induced by treatment with cART and isolation of CD4 positive cells, which eliminates actively infected cells (Fig 3A). Latent cells can then be reactivated using latency reversing agents, with reactivation often compared a “maximum” reactivation with positive control αCD3/αCD28 beads, which potently reverses latency in this model. β-catenin inhibitors ADV (100 nM) and PNU (100 μM) reactivated HIV NL4-3 from latency, quantified as the percent of p24-containing cells following drug treatment, above vehicle control in six of seven donors, albeit not significantly (p = 0.13 and 0.18 for ADV and PNU, respectively) (Fig 3B). ADV and PNU reactivation potential was 39% and 50% relative to “maximal reactivation” by αCD3/αCD28, which is comparable or exceeds the rate of other non-PKC agonist LRAs in this model (Fig 3C) [44, 45].

To date, primary HIV latency models have utilized replication-defective or laboratory adapted HIV strains [45]. To test the ability of this latency model to support a more clinical virus strain, as well as to test β-catenin inhibitors against a non-laboratory adapted virus, we infected cells in the T_CM_ model with transmitted/founder virus HIV REJO. We found that T_CM_ cells were less readily infected by HIV REJO, with an average infection of 2.0% and 7.7% of cells compared to 8.8% and 40.1% for NL4-3 at day 3 and day 6 post infection for 7 donors, respectively (representative donor shown in Fig 3A). HIV REJO infection also spread more slowly in these cells; with average replication rate, defined as the spread of infected cells between day 3 and day 6 post-infection, of 2.9 for REJO compared to 5.2 for NL4-3 (Fig 3D). This is likely due to the ability of CXCR4 utilizing NL4-3 to infect quiescent cells as compared to primary strains, which often require high levels of activation for infection. Despite lower levels of infection, the latency ratio, or the percentage of total infected cells that reactivate upon stimulation, is not different between HIV NL4-3 versus REJO, leading to lower but detectable latently infected populations in this model using a primary HIV strain (Fig 3E). We found that β-catenin inhibitor ADV (100 nM) significantly reactivated HIV REJO from latency above vehicle control in 7 of 7 donors (p = 0.028), at comparable levels to SAHA (1 μM) (Fig 3F). This corresponded to an average of 60% activity of ADV compared to αCD3/αCD28 beads, comparable to that of SAHA (58%) (Fig 3G). Significant loss in cell viability was observed in these cells when treated with β-catenin inhibitor ADV, but not PNU (S2A and S2B Fig). The increased toxicity of ADV observed in this model may be due to the prolonged culture of these primary cells in resting conditions and the anti-apoptotic role of β-catenin, whose protein level is decreased by ADV. These data demonstrate that inhibition of β-catenin can reactivate HIV strains from latency, including a clinical transmitted/founder virus, in primary CD4+ T cells from diverse donors.

### β-catenin pathway inhibition enhances the latency reversal of other LRAs

**Fig 4:**
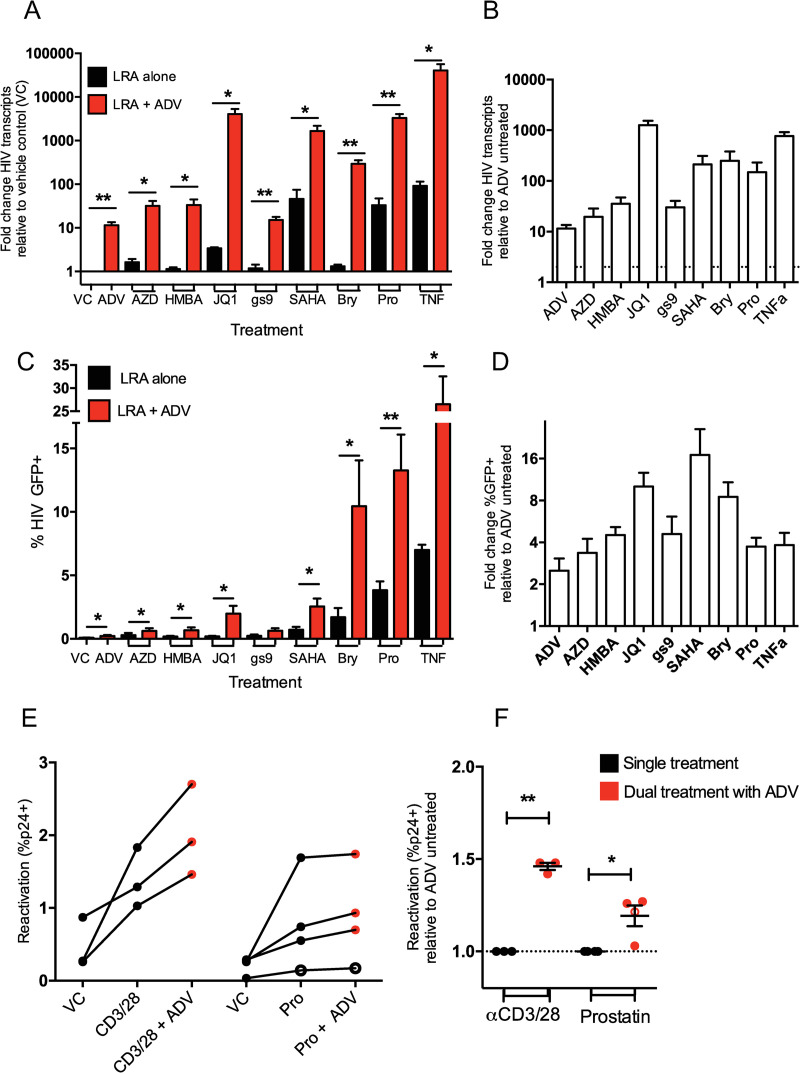
β-catenin pathway inhibition enhances HIV latency reversal of other drugs. (A) Treatment of J-Lat 8.4 cells with established LRAs alone (black) or in combination with 100 nM β-catenin inhibitor ADV (red) for 48 hours. Fold change in HIV *gag* RNA transcript levels over vehicle control (DMSO) is shown. (B) Fold change in HIV *gag* RNA levels for LRAs used in combination with ADV, normalized to HIV RNA levels when treated with the LRA alone. (C) Percent of cells positive for intracellular HIV GFP reporter after treatment with LRAs alone (black) or in combination with ADV (red). (D) Fold increase in cells positive for HIV protein (GFP) with ADV treatment, normalized to single LRA treatment. Columns indicate mean of 6 replicates with SEM error bars for (a-d). (E) Latent infection of HIV NL4-3 (closed circles) and REJO (open circles) was modeled in primary CD4+ T_CM_ cells from three to four donors and reactivated with αCD3/αCD28 beads and 1 μM prostatin alone (black) or in combination with 100 nM ADV (red) for 48 hrs. Percent reactivation (percent CD4- p24+ cells) is plotted for each donor with connecting lines. (F) Fold change in the percent of CD4 negative HIV p24 positive cells with combination ADV treatment, normalized to single treatment, is shown. Significance was determined using paired *t*-tests for all panels, * p<0.05, ** p<0.01.

Given that β-catenin inhibition can reactivate HIV latency, and that this pathway is distinct from other classes of existing LRAs, we sought to determine whether β-catenin could work additively or synergistically with other latency modulating agents. We tested eight known LRAs, HDAC inhibitor SAHA (1 μM), TLR-7 agonist gs-9620 (100 nM), BRD inhibitor JQ1 (1 μM), PKC agonists prostatin and byrostatin-1 (1 μM and 100 nM, respectively), SMAC mimetic AZD5582 (100 nM), P-TEFb activator HMBA (1 mM), and T cell stimulating cytokine TNF-α (10 ng/ml), alone or in combination with β-catenin inhibitor ADV (100 nM) in J-Lat 8.4 cells. ADV was chosen for combination testing over other β-catenin inhibitors due to its higher potency. We found that ADV treatment increased the levels of reactivated HIV transcripts in cells treated with all known latency reversing agents (Fig 4A). Each LRA was increased by at least the 12-fold induction rate of ADV alone, indicating the ability of ADV to have complementary reactivation with each drug tested (Fig 4B). Notably, some LRAs experienced increases in HIV RNA reactivation far exceeding the potential contribution of ADV alone, suggesting a potential synergistic effect of these drug classes, including prostatin, byrostatin-1, SAHA, TNF-α, and JQ1, with 149-, 250-, 211-, 771-, and 1257-fold increase in intracellular RNA following reactivation in combination with ADV, respectively (Fig 4B).

β-catenin inhibition also enhanced the ability of each LRA to induce reactivation of HIV proteins, as measured by detection of the J-Lat HIV GFP reporter (Figs 4C and S3A). Combination ADV treatment increased the negligible HIV protein reactivation of some drugs to more robust levels, such an increase of 0.2% to 2.0% of cells treated with JQ1, from 0.7% to 2.5% of cells treated with SAHA, and 1.7% to 10.4% of cells treated with bryostatin-1, despite ADV alone stimulating only 0.2% GFP positivity (Fig 4C). The ability of each LRA to reactivate HIV GFP was enhanced by at least 3.4-fold with ADV coadministration, with ADV alone increasing GFP expression 2.5-fold over vehicle control, suggesting the ability of β-catenin modulators to complement each class of LRA tested to induce HIV protein reactivation (Fig 4D), with modest loss of viability in dual treatments with the exception of TNF-α (S3B Fig). Notable LRAs which were highly enhanced by ADV treatment include bryostatin-1, JQ1, and SAHA, which had 8.5-, 10.0-, and 17.0-fold increase in HIV GFP reactivation (Fig 4D).

To formally query ADV’s ability to synergize with other LRAs, we employed a statistical test based on the Bliss definition of drug independence [46]. The Bliss independence model is commonly used to detect the synergistic action of drugs with differing mechanisms of action, including LRAs for combinatorial HIV latency reversal [30, 47]. We tested the ability of each LRA to act in synergy with ADV to induce both HIV RNA and protein. We find that ADV synergizes with every LRA class tested, with statistically significant synergy between ADV and AZD5582, HMBA, and gs-9620 for inducing HIV protein, and JQ1, SAHA, byrostatin-1, prostatin, and TNF-α for both HIV RNA and protein expression (Table 1).

**Table 1:** Bliss Independence Test for Synergy with β-catenin inhibitor ADV.

LRA	RNA *p*-value^a^	Protein *p*-value
AZD5582	0.11	5.85E-04
HMBA	0.15	3.46E-04
JQ1	1.61E-08	2.81E-05
gs-9620	0.25	1.62E-03
SAHA	7.04E-03	4.57E-04
Byrostatin-1	4.95E-08	2.05E-05
Prostatin	6.07E-05	4.17E-06
TNF-α	2.55E-03	5.94E-07

^**a**^p-value <0.05 indicates synergy

The combinatorial effect of β-catenin inhibition on latency reversal was also tested in the primary T_CM_ model. We found that ADV treatment increased reactivation of latent NL4-3 and REJO infection in all three to four donors treated with αCD3/αCD8 and prostatin (Fig 4E), as quantified by detection of intracellular p24 protein following reactivation. Together, ADV increased reactivation by αCD3/αCD8 stimulation by 1.5-fold (p = 0.0019) and PKC agonist prostatin by 1.2-fold (p = 0.041) (Fig 4F), with insignificant loss in cell viability in dual treatments, albeit with donor variability (S3C Fig). These data indicate that β-catenin inhibition can increase the latency reversing potential of diverse classes of LRAs, with synergistic effects in every LRA tested.

### Activation of β-catenin inhibits latency reversal

**Fig 5:**
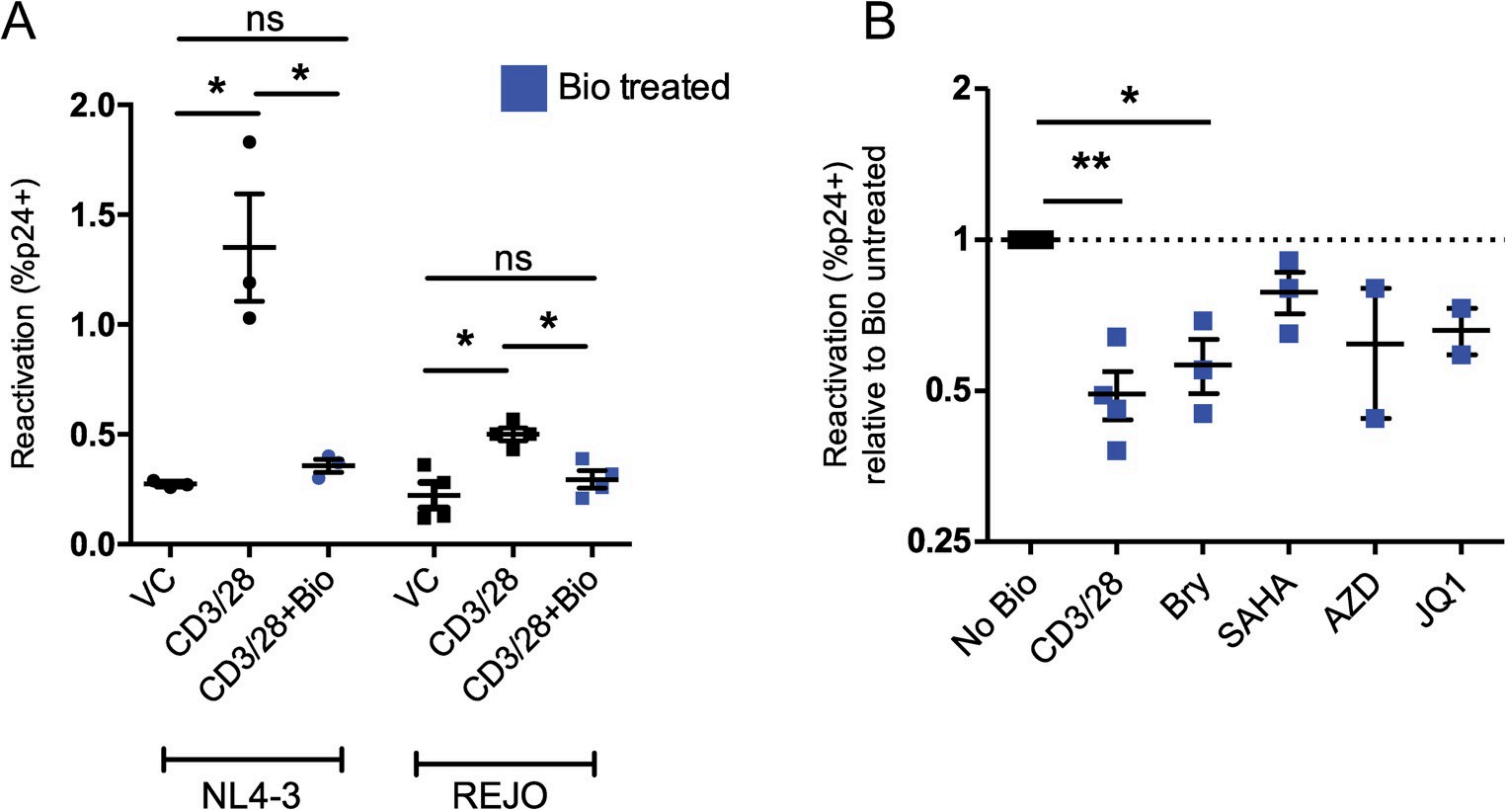
Activation of β-catenin inhibits HIV latency reversal. (A) 3–4 human donors were used to establish a primary T_CM_ model of HIV latency using HIV strain NL4-3 (left) or REJO (right). Latently infected cells were stimulated with αCD3/αCD8 activation beads alone (black) or in the presence of 2 μM Bio (blue). The percentage of CD4 negative HIV p24 positive cells is shown. (B) T_CM_ cells latently infected with HIV REJO were stimulated with the indicated drugs in combination with 2 μM Bio (blue). Reactivation as measured by the percentage of CD4 negative HIV p24 positive cells was normalized to reactivation with the drug in the absence of Bio. Mean and SEM of 3 donors is shown. Significance was determined using paired *t*-tests for all panels, * p<0.05, ** p<0.01.

Given the latency reversing effect of β-catenin inhibition, we addressed whether activation of β-catenin could inhibit latency reversal. We found that the β-catenin activating compound 6Bio (2μM) inhibited the latency reversing ability of αCD3/αCD28 activation beads to levels of vehicle control in primary T_CM_ cells latently infected with HIV NL4-3 and REJO, as measured by detection of intracellular p24 protein following reactivation, from 4.9-fold to 1.1-fold over vehicle for NL4-3 and from 3.3-fold to 1.6-fold over vehicle for REJO (Fig 5A), and without cellular toxicity (S4A Fig). Further, we found that the latency inhibiting effect of β-catenin activation was not specific to αCD3/αCD28, but rather reduced the HIV REJO latency reversal of several classes of LRAs, including bryostatin-1 (0.49-fold reactivation with 6Bio, p = 0.025), SAHA (0.55-fold with 6Bio, p = 0.11) in the T_CM_ model (Fig 5B), with modest effects on cellular viability, with the exception of SAHA treated cells (S4B Fig). These data demonstrate that β-catenin activation can robustly inhibit latency reversal through multiple pathways of viral reactivation and further suggests a role for β-catenin in the maintenance of HIV latency.

### Modulation of β-catenin enhances or inhibits latency reversal in primary HIV-infected patient samples ex vivo

**Fig 6:**
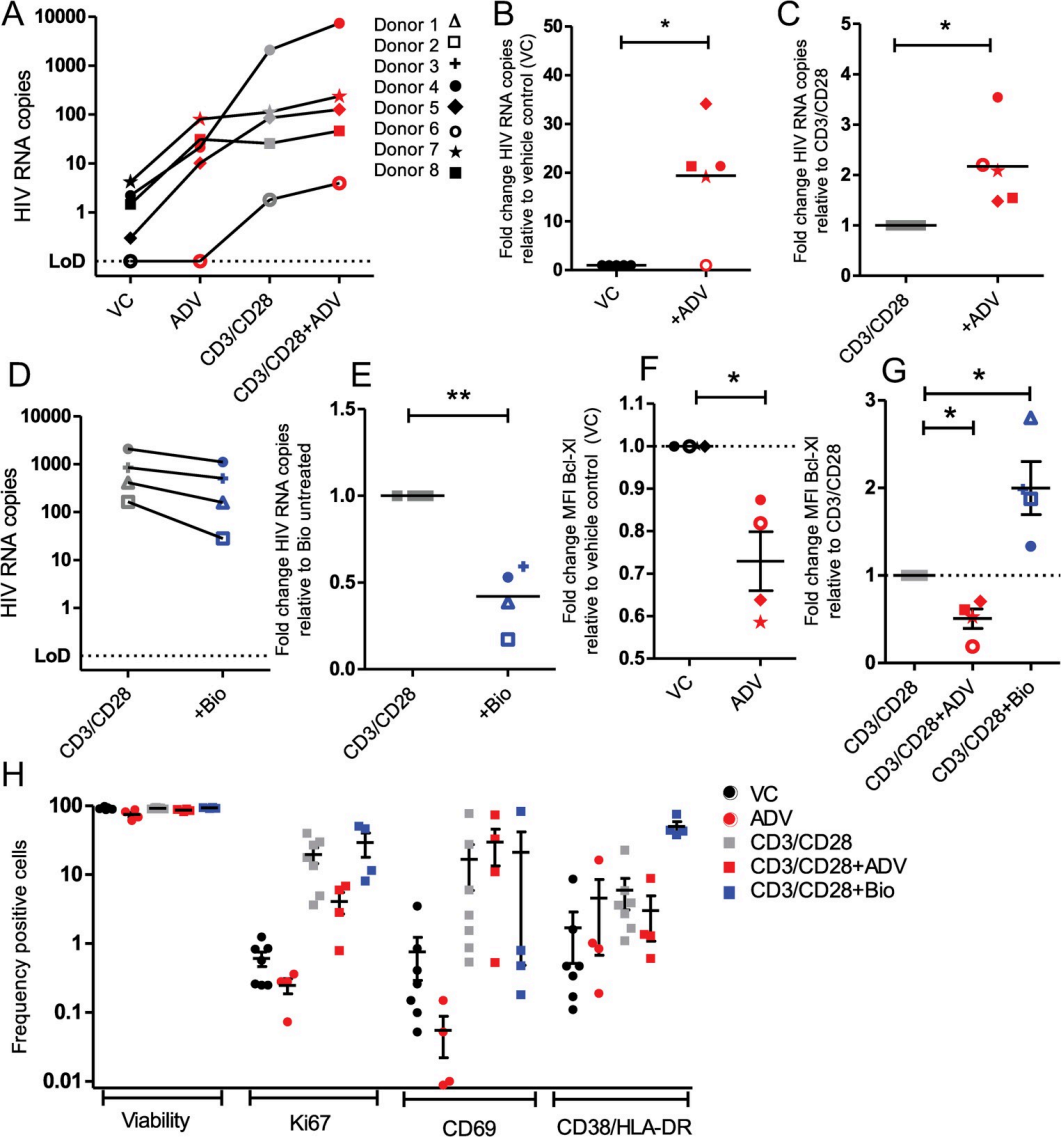
β-catenin modulation impacts HIV latency reversal in cells from HIV-infected virally suppressed individuals. (A) CD8-depleted PBMCs from n = 5 HIV positive donors on suppressive cART therapy were treated for 48 hours with 50 nM β-catenin inhibitor ADV, αCD3/αCD28 T-cell activating beads alone or combined with 50 nM ADV. Extracellular (released virions, right) HIV RNA copies were quantified. Absolute RNA copy numbers in vehicle control and ADV treated cultures are shown, with lines connecting samples from the same donor. Symbols corresponding to donors in Table 2 are used for panels a-g. (B) Fold change of RNA copies in ADV treated cultures over vehicle control are shown from released virus. (C) Fold change of RNA copies in ADV co-treated cultures over αCD3/αCD28 single treatment. (D) As in (A), cells were treated with αCD3/αCD28 beads alone or combined with 2 μM 6Bio. HIV RNA quantities are shown with lines connecting cultures from the same donor. (E) Fold change of HIV RNA copies in 6Bio co-treated cells over αCD3/αCD28 single treatment. (f-g) Downstream target of β-catenin, Bcl-xL, was quantified by flow cytometry in cells treated with ADV, αCD3/αCD28, or αCD3/αCD28 with ADV/6Bio, to confirm the modulation of β-catenin by these drugs. Fold change in mean fluorescence intensity of Bcl-xL is shown compared to vehicle control or αCD3/αCD28 treated cells, for single or dual treated cells, respectively. (H) Viability and T cell activation markers in cells following drug treatments were quantified by flow cytometry. The proportion of CD3+ CD4+ T cells expressing Ki67, CD69, CD38/HLA-DR, or LIVE/DEAD stain are plotted for cells treated with the indicated treatments. Significance was determined using paired *t*-tests for all panels, * p<0.05, ** p<0.01.

Given the ability of small molecules targeting β-catenin to modulate HIV latency in T cell lines and the primary T_CM_ latency model, we next tested β-catenin modulation in latently infected cells from HIV positive donors on suppressive cART (Table 2). We found that ADV treatment (50 nM) of CD8-depleted PBMCs from virally suppressed donors increased reactivated virus released into supernatant in 4 of 5 donors, with 19.1-fold increase in released virus (p = 0.026), as measured by quantification of HIV RNA in the supernatant of cultured cells (Fig 6A and 6B). ADV similarly increased the reactivation potential of αCD3/αCD8 beads in all 5 of 5 donors, increasing released virions by 2.1-fold (p = 0.0343) (Fig 6A and 6C). β-catenin activation by 6Bio inhibited reactivation by αCD3/αCD28 beads in 4 of 4 donors, resulting in mean 0.42-fold levels of released virus (p = 0.0085) (Fig 6D and 6E). We found that 50 nM ADV and 2 μM Bio was sufficient to significantly decrease or increase, respectively, levels of downstream β-catenin target protein Bcl-Xl without inducing cell toxicity in these primary HIV donor cells when used in combination with αCD3/αCD28 beads (Fig 6F, 6G and 6H); ADV treatment alone caused a mean 17% loss of viability compared to vehicle control (Fig 6H). These data show that β-catenin modulation can alter HIV latency in the most physiological model outside of human subject trials.

**Table 2:** Clinical characteristics of HIV virally suppressed blood donors.

Donor	CD4 Date	CD4 count, absolute (%)	Viral Load (VL) Date	VL copies/ml	Year of HIV Dx	Years on Suppressive ART^b^	Age	Race^a^	Sex
1	11/5/2020	1247 (43%)	4/27/2021	<40	2017	4	24	W	M
2	4/27/2021	809 (57%)	4/27/2021	<40	2007	6	49	B	M
3	4/27/2021	787 (30%)	4/27/2021	<20	1999	>10	51	H	M
4	10/22/2020	1082 (47%)	5/10/2021	<40	1997	>10	67	W	M
5	5/12/2021	726 (23%)	5/12/2021	<40	2015	4	53	H	M
6	5/18/2021	515 (26%)	5/18/2021	<40	2006	5	44	B	M
7	9/16/2020	690 (34%)	5/25/2021	<20	1994	>11	57	H	M
8	6/1/2021	198 (15%)	6/1/2021	<40	2002	6	37	B	F

^a^White (W), Black (B), Hispanic (H)

^b^Number of years since first undetectable VL, “>” if unknown ART start date

A main concern of early LRAs was their global T cell activation, which can result in significant off-target effects and undesirable side-effects in patients. We found that treatment with ADV did not induce markers of T cell proliferation/activation Ki67, CD69, CD38, or HLA-DR, suggesting that β-catenin inhibitors induce HIV reactivation in the absence of T cell activation (Fig 6H). ADV treatment even reduced levels of Ki67 expression when co-administered with αCD3/αCD8 beads in all donors from mean 19% to 4% (p = 0.1099), suggesting a potential benefit to combining β-catenin inhibitors with LRAs which that have T cell stimulating effects (Fig 6H). Treatment with 6Bio had variable effects on activation markers, having no effect on Ki67, decreasing CD69 in 3 of 4 donors, and increasing CD38/HLA-DR levels (Fig 6H). This is likely because 6Bio activates β-catenin through GSK3β inhibition, which negatively regulates T cell responses [48]. CD8-depletion of donor PBMCs was chosen to maximize latent cell retention, however, one caveat of using CD8-depleted PBMCs is that non-CD4+ T cells factors could secrete factors that impact latency reversal.

## Discussion

HIV latency is the main obstacle to an HIV cure and understanding the mechanisms that underlie HIV latency will be key to developing a sterilizing or functional cure strategy. The mechanism of HIV latency is complex, involving numerous viral and host proteins and pathways. Importantly, each pathway involved in HIV latency maintenance provides a potential target for HIV cure strategies. Here, we describe a novel pathway for the control of HIV latency through the transcriptional coregulator β-catenin. We found that β-catenin modulation can alter HIV latency in latent T cell lines, in a T_CM_ primary cell model of HIV latency, notably using a clinical virus in this model for the first time, and further in latently infected cells from cART-controlled HIV+ individuals. These results both confirm a novel mechanism of HIV latency and reveal a new class of drugs that may be used to modulate HIV latency.

**Fig 7:**
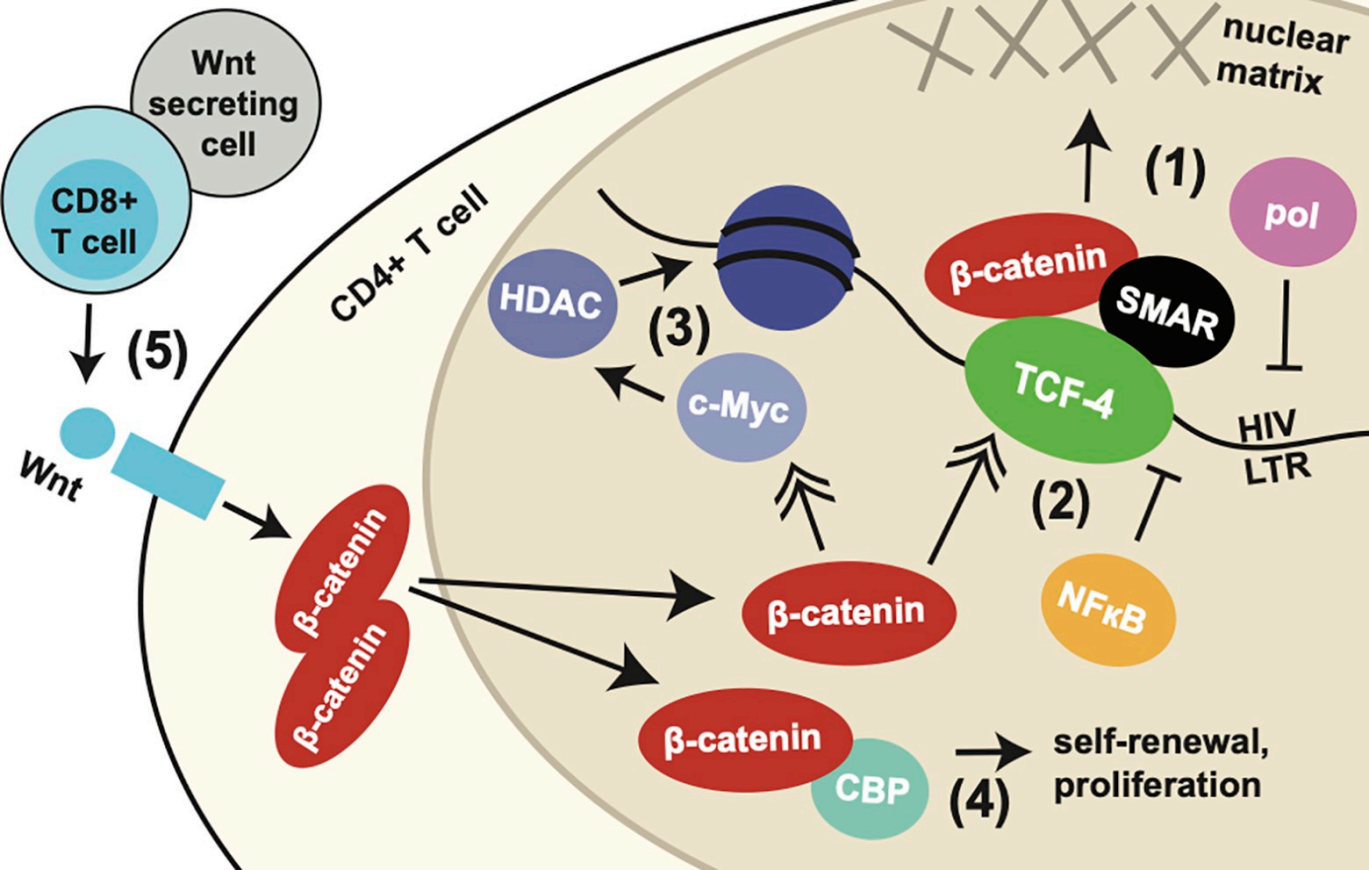
Integrated model of potential mechanisms of HIV transcription and latency modulation by β-catenin. Schematic demonstrating multiple potential mechanisms by which β-catenin may modulate HIV transcription and latency, based on integrated findings from previous studies and data presented here. (1) β-catenin and TCF-4 form a complex with nuclear matrix-associated protein SMAR1 at the HIV LTR just upstream of the transcriptional start site and Sp-1, NFκB, and AP-1 binding sites. This complex pulls the HIV LTR towards the nuclear matrix, occluding access of RNA polymerase [35]. (2) β-catenin positively regulates levels of TCF-4, which has been shown to block binding and transcriptional regulation of NFκB at the HIV LTR [70]. (3) β-catenin further regulates c-Myc levels, which recruit HDAC enzymes, resulting in the viral promoter being more densely packed in chromatin [51]. (4) β-catenin also mediates self-renewal and cell proliferation of memory T cells through CBP, which may contribute to perpetuating the reservoir of latently infected cells [60], (5) A source of β-catenin signaling are CD8+ T cells, which secrete Wnt proteins resulting in stimulation of the Wnt/β-catenin pathway in CD4+ T cells, which culminates in accumulation of β-catenin in the cytoplasm and translocation to the nucleus [33]. This may explain the observed role of CD8+ T cells in maintaining HIV latency. Notably, other cells may serve as a source of Wnt proteins and β-catenin pathway modulating factors.

We find that β-catenin acts directly at the HIV LTR via transcription factor TCF-4, which binds at an LTR site rich in transcription factor binding sites just upstream of NF-κB and AP-1 binding sequences and ~140 bp upstream of the transcriptional start site. However, we found that this binding site did not fully account for the ability of β-catenin to inhibit HIV transcription. Other putative TCF-4 binding sites have been observed in the HIV LTR; binding of TCF-4 to these sites has been demonstrated by chromatin immunoprecipitation, and one of these sites was shown here to have the potential to inhibit HIV LTR activity [35]. However, these sites are less intact among HIV strains and further from or downstream of the transcription start site, suggesting a likely smaller role of these sites. Inhibition of HIV transcription that is independent of the -143 LTR binding site could be mediated by other genes that are regulated by β-catenin. For example, β-catenin positively regulates TCF-4 levels, which have in turn been shown to inhibit NF-κB binding to the HIV LTR [49, 50]. β-catenin also positively regulates c-Myc gene expression, which has been shown to recruit HDAC to the HIV LTR [51, 52]. Thus, the activity of β-catenin in HIV transcription inhibition and latency maintenance may be enhanced by the other genes controlled by β-catenin (integrated mechanisms summarized in Fig 7).

The identification of this unique pathway is important for its potential complementary or synergistic effect with existing classes of LRAs. Given the initial failure of LRA monotherapy trials and the partial activity of LRAs in heterogeneous cell populations, it is now largely accepted that substantial latency reversal *in vivo* will require a combination of potent LRAs targeting diverse pathways [53]. Importantly, we found statistically significant synergistic action at the protein level of β-catenin inhibitors with each class of LRA tested, suggesting that combination therapy of β-catenin inhibitors could benefit T cell activators, epigenetic modifiers / HDAC inhibitors, TLR agonists, BRD inhibitors, PKC agonists, non-canonical NF-κB stimulators, pTEF-b modulators, and potentially others. β-catenin inhibitors could be added to combination therapies which have already been identified to work synergistically, such as PKC agonists with HDAC inhibitors or BRD inhibitors [30, 54, 55]. As mentioned, β-catenin regulates gene expression of many genes, which may contribute to the synergistic effect with some LRAs. For example, inhibition of β-catenin would result in decreased c-Myc, which could decrease HDAC binding to the LTR, contributing to the activity of HDAC inhibitors [51, 52]. Alternatively, β-catenin activation could promote HDAC recruitment, complementing its own transcriptional silencing activity in efforts to promote deep latency. Indeed, others have found that β-catenin levels can affect the functionality of HDAC inhibitors in a cancer study [56]. Further, inhibition of β-catenin, which regulates cell cycle, could help dampen the cell proliferative qualities of PKC agonists and T cell activators, which may result in undesirable inflammatory side effects in patients and which may have the potential to expand the HIV reservoir by promoting clonal expansion of latently infected cells. Indeed, we showed that β-catenin inhibition showed a trend towards lowered T cell proliferation and activation markers when combined with T cell stimulating treatments, suggesting that β-catenin inhibitors could help suppress undesirable T cell activation in combination therapies. As such, the addition of β-catenin modulators to the existing arsenal of latency modifying agents provides new opportunities for combination therapies towards comprehensive latency reversal or promotion of deep latency.

β-catenin may be considered a relatively risky target for HIV latency due to its many cellular functions and association with cell survival and cancer. Indeed, mutations in β-catenin encoding gene *CNNTB1* and other genes which result in accumulation of β-catenin are associated with malignancies, however, it is unclear if some cases of increased β-catenin are a cause of the malignancy or a response to it. For example, a mutation in APC is linked to colon cancer, this mutation leads to unchecked expression of β-catenin and the cancer is due to APC mutation which in turn leads to sustained over expression of β-catenin overtime that mediates enhanced cell turnover. Additionally, evidence suggests that activation of the Wnt/β-catenin pathway can actually inhibit melanoma proliferation and metastasis, suggesting a complex role of this pathway in cancer [57]. Several clinical trials targeting the Wnt/β-catenin pathway have been performed with promising safety data, although bone resorption side effects have been observed with some inhibitors due to the key role of the Wnt pathway in skeletal bone maintenance [58, 59]. Direct β-catenin inhibition has not yet been included in cancer drug trials. This may be due to the paucity of small molecules that bind β-catenin directly, most inhibitors of β-catenin activity rather target the upstream Wnt signaling pathway, although a few molecules with the potential to bind β-catenin have recently been identified, including PNU-74654. The safety of utilizing small molecule inhibitors or agonists of β-catenin would need to be carefully evaluated *in vivo*. In context of HIV cure strategies, the use of β-catenin modulators is envisioned to be transient and not long-term and as such any side effects may be relatively limited. At least in a SIV macaque study, inhibition of β-catenin was tolerated for at least 12 weeks [60].

Small molecule activators of β-catenin also often target upstream targets of the Wnt/β-catenin pathway. The compound used here, 6Bio, is a GSK-3 inhibitor which results in β-catenin activation [61, 62]. 6Bio has also been shown to have pan-JAK inhibitory effects at the concentration used in this study [63]. GSK-3 and JAK are involved in numerous cellular pathways, some of which could impact HIV latency reversal. Thus, the specificity of β-catenin activation in inhibiting latency reversal would need to be confirmed.

Several latency reversing agents that were shown to be effective *in vitro* and *ex vivo* have failed to show significant effects in preclinical models and/or clinical trials. Thus, testing the ability of β-catenin modulation in an animal model of HIV or SIV latency will be critical in assessing its potential for cure strategies. Notably, one study which targeted the Wnt/β-catenin pathway in SIV infected rhesus macaques by treating with a small molecule that disrupts the interaction between CBP and β-catenin, PRI-724, showed that while this molecule did reduce the proliferation of central memory and stem cell memory T cells, which contribute to the viral reservoir, the overall size of the viral reservoir in these animals was unchanged [60]. However, as previously discussed, effective latency modulating strategies will likely require a combination of agents targeting multiple HIV latency pathways. Thus, the utility of β-catenin modulators in preclinical or clinical experiments may not be apparent when tested in isolation. Notably, the β-catenin pathway inhibitor was found to be well-tolerated in these animals, even when given daily for 12 weeks, with no changes to hematological parameters, liver and kidney functions, and no weight loss or clinical adverse events when given at 20 or 40 mg/kg/day [60].

Importantly, our results may also provide a mechanism for the observation that CD8+ T cells promote viral latency. Multiple studies of SIV infected macaques have recently found that CD8 depletion can significantly enhance latency reversal [24, 34, 64]. In one study, CD8-depletion increased the success of SMAC mimetic AZD5582 at reversing latency in ~60 to 100% of animals [23, 34] and in another it increased the success of IL-15 superagonist N-803 from 0% to 100% of animals [24]. A subsequent study by this group revealed that the observed latency-supporting activity was provided by non-HIV-specific CD8+ T cells and a non-cytolytic mechanism, recalling an early observation that CD8+ T cells are capable of suppressing HIV through transcriptional inhibition [65, 66]. Indeed, multiple *in vivo* studies have observed the ability of CD8+ T cells to suppress HIV in a non-cytolyic fashion [67, 68]. Our group recently discovered that the non-cytolytic HIV-suppressing capacity of CD8+ T cells is mediated by the secretion of Wnts and subsequent activation of the Wnt/β-catenin pathway in CD4+ T cells [33]. Combined with our results here, this would suggest a mechanism by which CD8+ T cells secrete Wnts, which trigger the Wnt signaling cascade in CD4+ T cells leading to the upregulation of β-catenin, which promotes the transcriptional silencing of HIV and maintenance of HIV latency. This would also suggest that the latency-reversing effect of CD8-depletion may be accomplished with a more targeted suppression of β-catenin, making the elimination of CD8-T cell blockade to latency reversal more clinically feasible.

Collectively, our study reveals the β-catenin pathway as a novel player in the mechanism of HIV latency to inform a new class of reagents which may be used for the design of a combination HIV cure therapy. Specifically, the ability to target β-catenin pathway through its activation or inhibition to promote HIV latency or reversal of latency, respectively, places β-catenin at the interface of both shock and kill and block and lock cure strategies. As such, this represents one of the few pathways described that can be manipulated in a way to achieve a specific outcome for HIV strategy depending on how it is manipulated.

## Materials and methods

### Ethics statement and human blood samples

Research involving human subjects was conducted in accordance with institutional (IRBL06080703) and U.S. government guidelines on human research. Whole blood was collected from both healthy HIV seronegative donors and HIV-positive patients at Rush University Medical Center. Written informed consent was obtained from all participants prior to blood donation, and this study was approved by the Institutional Review Board of Rush University Medical Center and the Cook County Health and Hospitals System.

### LTR luciferase reporter assay

Luciferase reporter plasmids with TCF/LEF binding site deletions were constructed as previously described [35]. Briefly, LTR was amplified from HIV-1 BAL infected PBMCs and cloned into pGL4.19 plasmid (Promega). Wildtype LTR was subjected to site-directed mutagenesis using the QuikChange multikit (Stratagene) to derive the mutants harboring a deletion in the TCF-4 binding sites at positions -143, +186, or -413 and +186, relative to the transcriptional start site, mutations were confirmed by Sanger sequencing.

For primary CD4+ T cells, cells were enriched from whole blood using RosetteSep Human CD4+ T Cell Enrichment Cocktail (StemCell Technologies) according to manufacturer’s protocol. Cells were cultured in RPMI supplemented with 10% fetal bovine serum, 1X penicillin/streptomycin, and 30 U/ml IL-2 then activated for 24 hours using Dynabeads Human T-Activator CD3/28 beads (Gibco) at a 1:1 bead to cell ratio. One million activated cells were nucleofected using the 4D-Nucleofector system (Lonza) and P3 Primary Cells kit (Lonza) using the 20μl Nucleocuvette strip, 0.8μg plasmid DNA, and pulse code E0-115. Cells were harvested in 1X passive lysis buffer (Promega) 24 hours post-nucleofection, and luciferase reporter activity was determined using a dual-luciferase reporter assay (Promega) and a single-injector luminometer. Relative light units were normalized to total protein concentration of the lysate (μg/ml), as quantified by a Pierce bicinchoninic acid (BCA) protein assay kit (Thermo Fisher Scientific).

### TCF binding site mutant HIV infectious molecular clones and infections

HIV-1 REJO infectious molecular clone plasmid (pREJO) was obtained through the NIH AIDS Reagent Program (Division of AIDS, NIAID, NIH). A fragment of the 5’ LTR surrounding the -143 TCF binding site was removed via restriction digest using enzymes MluI and BssHII (New England Biolabs). Wildtype or TCF binding site mutant sequences spanning the excised LTR fragment with 20 base pair overhangs were synthesized (Integrated DNA Technologies) and reassembled to pREJO using NEBuilder HiFi DNA Assembly kit (New England Biolabs) then propagated in MAX Efficiency Stbl2 competent cells (Invitrogen). Sequence of the entire infectious molecular clone was confirmed by Sanger sequencing, with resulting sequences matching the original construct (REJO-WT) or containing a mutated TCF binding site in the 5’ LTR (REJO-MUT). Viruses were produced via transient transfection of HEK293T cells using Fugene 6 Transfection Reagent (Promega) and virus titers (infectious units (IU)/ml) were determined using β-galactosidase staining of TZM-bl cells. Primary CD4+ T cells were activated using Dynabeads Human T-Activator CD3/28 beads (Gibco) at a 1:1 bead to cell ratio for 24 hours, then infected with HIV REJO-WT or HIV REJO-MUT viruses at a multiplicity of infection (MOI) of 0.01 by spinoculation of cells at 1,200xg for 2 hours. Following spinoculation, cells were washed of virus-containing media and plated in complete RPMI supplemented with 30 U/ml IL-2 for 24 hours. For PBMC infections, cells were activated using 5 μg/ml PHA-P (Sigma-Aldrich) for 24 hours, then infected 3-days post-activation via spinoculation and cultured in complete RPMI with 30 U/ml IL-2 for 7 days. Cellular RNA was harvested using the RNeasy Mini Kit (QIAGEN) and DNA was removed using on-column RNase-Free DNase Set (QIAGEN). cDNA was produced using qScript cDNA SuperMix (Quantabio) and HIV RNA was quantified using HIV *gag* primers 5’-CCC AGA AGT GAT ACC CAT GTT-3’ (forward) and 5’-GCT TCC TCA TTG ATG GTC TCT-3’ (reverse) and PowerUp Sybr Green Master Mix (Applied Biosystems) on a Quantstudio 7 Flex Real-Time PCR System (Applied Biosystems). Relative CT values were normalized using the housekeeping gene GAPDH.

### HIV reactivation from latent cell lines

OM-10.1 and J-Lat (8.4) cells were obtained from the NIH AIDS Reagent Program (Division of AIDS, NIAID, NIH) and maintained in RPMI supplemented with 10% FBS and 1X penicillin/streptomycin. β-catenin inhibitors PNU-74654, adavivint (SM04690), ICG-001, and iCRT14 were obtained from Selleckchem and reconstituted in DMSO. LRAs TNFα (10 ng/ml, Gibco), SAHA (Vorinostat) (1 μM, abcam), Prostatin (1 μM, Sigma), Bryostatin-1 (100 nM, Sigma), JQ1 (1 μM, abcam), AZD5582 (100 nM, Selleckchem), and GS-9620 (100 nM, Selleckchem) were reconstituted in DMSO, with the exception of Hexamethylene bisacetamide (HMBA) (1 mM, abcam), which was reconstituted in water and used to treat cells at the indicated final concentrations. DMSO was used as a vehicle control at the same final concentration as drug treated wells. For drug treatments of β-catenin inhibitors and LRAs, 500,000 cells were plated in 0.5 mL media and incubated for 48 hours with drug solutions. When two drugs were used in combination, both drugs were added simultaneously. Cells were harvested for RNA or RNA and flow cytometry for OM-10.1 and J-Lat cells, respectively. HIV *gag* transcripts were quantified as described above. To confirm the suitability of GAPDH as a reference gene for relative quantification of HIV transcripts in latency reversal experiments, we quantified an additional housekeeping gene, 18S RNA, and found that GAPDH CT were stable between vehicle control and β-catenin inhibitor treated J-Lat and OM-10.1 cells (S1C Fig). GAPDH protein levels were also stable across drug treatments as detected by Western blot (S1B Fig). For flow cytometry, J-Lat cells were fixed in 1% paraformaldehyde (PFA) and run on a BD LSRFortessa flow cytometer.

To confirm the β-catenin pathway inhibition of small molecule β-catenin inhibitors in J-Lat 8.4 cells, cells were transduced with a lentivirus containing TOPflash, a β-catenin pathway reporter plasmid. Briefly, cells were infected overnight with lentivirus containing 7TFP (Addgene), produced by calcium phosphate transfection of HEK cells (psPAX2, pMD2.G, Addgene), then maintained in media containing 2 μg/mL puromycin. Cells were treated with β-catenin inhibitors for 48 hours, then luciferase was quantified as described above. Additionally, cells were treated for 48 hours, lysed in RIPA buffer, protein quantified by BCA assay, and 15 μg total protein was probed for β-catenin (Sigma, 1:20,000 antibody dilution), c-Myc (abcam, 1:2,000), and housekeeping protein GAPDH (Sigma, 1:25,000) by Western blot using standard techniques.

### siRNA knockdown of β-catenin

ON-Target*plus* Smartpool siRNAs (Dharmacon) specific for β-catenin and scrambled siRNA controls were nucleofected into J-Lat cells using the SE Cell Line kit and the 4-D Nucleofector System (Lonza). 300 nM siRNA was nucleofected into cells using program CL-120. Cells were harvested 24 hours post nucleofection. Viability was determined using trypan blue staining and TC20 Automated Cell Counter (BioRad). Intracellular HIV *gag* was quantified as above. Levels of *CTNNB1*, the gene encoding β-catenin, was quantified as above. Nucleofection efficiency was determined using the identical nucleofection protocol with 2 μg of mScarlet expression plasmid and measuring the percent of live cells expressing mScarlet by Fixable LIVE/DEAD Aqua stain (Invitrogen) and flow cytometry. The mean of two replicate mScarlet nucleofections was used for normalization.

### T_CM_ primary latency model

The T_CM_ model of HIV latency was followed according to Macedo et al. with minor modifications [44]. Briefly, naïve CD4 T cells were isolated from healthy donors using EasySep Human Naïve CD4+ T Cell Isolation Kit II (StemCell Technologies) then activated for 3 days with Dynabeads Human T-Activator CD3/28 beads (Gibco) in the presence of 10 ng/ml TGF-β1 (abcam), 2 μg/ml anti-human IL-12, and 1 μg/ml anti-human IL-4 antibody (R&D Systems). On day 7 post-activation, one fifth of total cells were infected via spinfection at an MOI of 1 and 0.3 for HIV-REJO and HIV-NL4-3, respectively. Following spinfection, three fifths of the remaining uninfected cells were added back to the spinoculated cells and one fifth was kept as an uninfected control. Six days following infection (d13), cells were treated with cART (1 μM raltegravir and 0.5 μM nelfinavir). CD4-positive cells, containing latently infected and uninfected cells, were isolated four days later (d17) using Dynabead CD4 Positive Isolation Kit (Invitrogen). 200,000 to 500,000 cells were stimulated with drugs as indicated for 48 hours in the presence of cART and harvested (d19) for determination of reactivation, as measured as the percentage of p24 positive CD4 negative cells. Cells were monitored via flow cytometry for viability, CD4, and p24 expression at days 10, 13, 17 and 19 using Fixable LIVE/DEAD Aqua stain (Invitrogen), anti-CD4-APC (Life Technologies), and KC57-RD1 PE (HIV p24, Beckman Coulter). Flow cytometry was performed on a BD LSRFortessa using FACSDiva software (Becton Dickinson) and analyzed using FlowJo software v10.7.1.

### Reactivation of latent HIV from virally suppressed donors

Blood was drawn from HIV positive patients on suppressive cART for at least 4 years (Table 2). CD8+ depleted PBMCs were isolated using the RosetteSep Human CD8 depletion cocktail (StemCell) and suspended in RPMI supplemented with 30 U/ml IL-2 and antiretrovirals raltegravir (1 μM) and nelfinavir (0.5 μM). Cells were immediately treated with Dynabeads Human T-Cell Activator CD3/28 beads (Gibco) and/or small molecule drugs and cultured for 48 hours, with around 10 million cells per treatment. HIV was quantified using a previously described highly sensitive semi-nested RT-qPCR [69]. Briefly, released virions were harvested using the QIAmp Viral RNA Mini Kit (QIAGEN) following concentration from the culture supernatant using Lenti-X Concentrator (Takara Bio). HIV was then reverse transcribed using SuperScript IV First Strand Synthesis System (Invitrogren), subjected to 16 cycles of amplification using Platinum II Taq Hot-Start DNA Polymerase (Invitrogen), and quantified using nested primers by q-PCR as described [69]. Serial dilutions of RNA isolated from culture supernatant of HIV IIIB infected cells and quantified using plasma viral load standards obtained from the NIH AIDS Reagent Program (Division of AIDS, NIAID, NIH) were amplified simultaneously and used to quantify absolute copies of HIV RNA. The expression of activation markers was measured by flow cytometry using the following panel: Fixable LIVE/DEAD Aqua stain (Invitrogen), CD3-Pacific Blue (BD Biosciences), CD38-BV786 (BD Biosciences), CD69-FITC (BD Biosciences), HLA-DR-PerCP (BD Biosciences), Ki-67-AF700 (BD Biosciences), Bcl-xL-PE (ThermoFisher Scientific).

### Statistics

Statistical analysis was performed using Prism software v5.00 (GraphPad). Variables were compared using paired or unpaired *t*-test, where appropriate and as indicated in figure legends. All tests were two-tailed, with a *p* value <0.05 considered significant. Statistical determination of LRA synergy based on the Bliss independence model was performed as described [46].

## Supporting information

S1 Fig Supporting information: Inhibition of the β-catenin pathway reactivates HIV in cell lines. J-Lat 8.4 cells were treated with known latency reversing agents TNFα, SAHA, or β-catenin inhibitors PNU-74654 (red), adavivint (blue), and ICG-001 (orange) for 48 hours, as in Fig 2B. (A) Inhibition of β-catenin activity following drug treatment was tested in J-Lat cells stably transduced with TOPFlash, a reporter plasmid containing TCF-4 binding sites upstream of a promoter and luciferase gene. RLUs were normalized to total protein, as quantified by BCA assay, and compared to vehicle treated cells. (B) Modulation of β-catenin and downstream target c-Myc protein levels was quantified by Western blot of 15μg J-Lat cell lysate; corresponding β-catenin (C) and c-Myc (D) abundance were quantified by densitometry in ImageJ and compared to vehicle control (mean of duplicate). Toxicity of the drug treatments in OM-10.1 (E) and J-Lat (F) was evaluated in three independent replicates by LIVE/DEAD red dead cell viability staining and flow cytometry. (G) Geometric mean fluorescence intensity in HIV GFP+ cells following drug treatments are shown. (H) Nucleofection efficiency of siRNA knockdown in Fig 2D was determined using mScarlet reporter plasmid and flow cytometry, average of duplicate reactions was used for normalization. (I) Three replicate treatments of OM-10.1 and J-Lat cells were quantified for two housekeeping genes, GAPDH and 18S RNA, to confirm the stability of GAPDH across treatments. GAPDH CT values (delta CT [GAPDH-18S]) were compared between vehicle control and β-catenin inhibitor treated J-Lat and OM10.1 cells. Significance was determined using paired *t*-tests for all panels, * p<0.05, ** p<0.01, *** p<0.001.
(TIF)

S2 Fig Supporting information: β-catenin pathway inhibition reactivates two HIV strains in a primary T_CM_ model of HIV latency. Toxicity of the drug treatments in NL4-3 (A) and REJO (B) infected cells were evaluated in each donor by LIVE/DEAD aqua dead cell viability staining and flow cytometry. Significance was determined using paired *t*-tests for all panels.
(TIF)

S3 Fig Supporting information: β-catenin pathway inhibition enhances HIV latency reversal of other drugs. (A) Geometric mean fluorescence intensity for HIV GFP, corresponding to experiment and GFP percentages shown in Fig 4C. Significance was determined using paired *t*-tests, * p<0.05, ** p<0.01. (B) Toxicity of the drug treatments were evaluated in two independent replicates by LIVE/DEAD red dead cell viability staining and flow cytometry. (C) Toxicity of the drug treatments in Fig 4E were evaluated in each donor by LIVE/DEAD aqua dead cell viability staining and flow cytometry and tested for significance using paired *t*-tests.
(TIF)

S4 Fig Supporting information: Activation of β-catenin inhibits HIV latency reversal. (A-B) Toxicity of the drug treatments in Fig 5 were evaluated in each donor by LIVE/DEAD aqua dead cell viability staining and flow cytometry. Significance was determined using paired *t*-tests for all panels, * p<0.05.
(TIF)

S1 Table Supporting information: All data used to generate Figs 1–7 and S1–S4. (XLSX)
