# Supplementary figures and images for: β-catenin regulates HIV latency and modulates HIV reactivation
Source: PLoS Pathog. 2022 Mar 7;18(3):e1010354. doi: 10.1371/journal.ppat.1010354 (PMC8939789; doi:10.1371/journal.ppat.1010354)

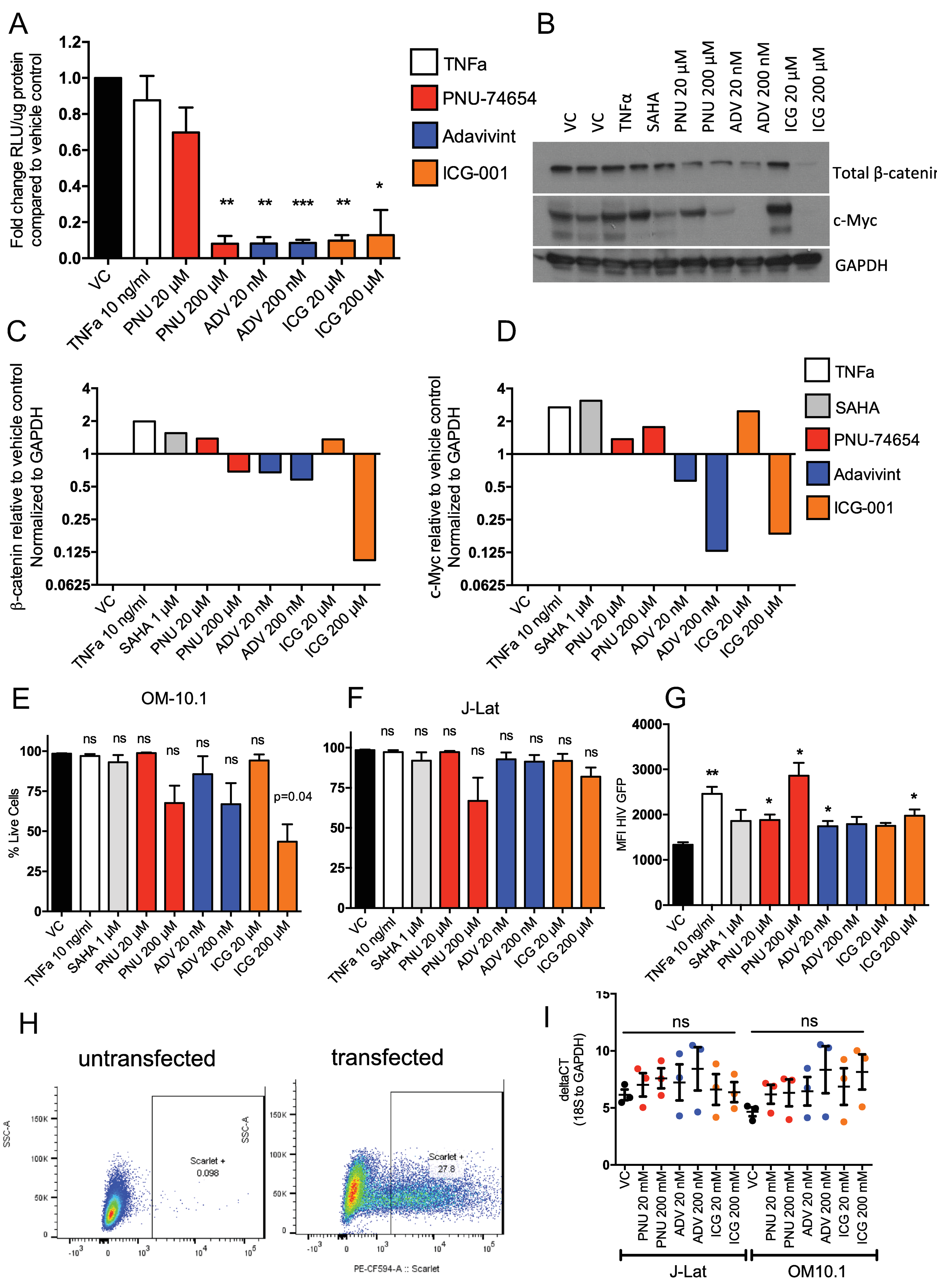

Supplement: S1 Fig — J-Lat 8.4 cells were treated with known latency reversing agents TNFα, SAHA, or β-catenin inhibitors PNU-74654 (red), adavivint (blue), and ICG-001 (orange) for 48 hours, as in Fig 2B. (A) Inhibition of β-catenin activity following drug treatment was tested in J-Lat cells stably transduced with TOPFlash, a reporter plasmid containing TCF-4 binding sites upstream of a promoter and luciferase gene. RLUs were normalized to total protein, as quantified by BCA assay, and compared to vehicle treated cells. (B) Modulation of β-catenin and downstream target c-Myc protein levels was quantified by Western blot of 15μg J-Lat cell lysate; corresponding β-catenin (C) and c-Myc (D) abundance were quantified by densitometry in ImageJ and compared to vehicle control (mean of duplicate). Toxicity of the drug treatments in OM-10.1 (E) and J-Lat (F) was evaluated in three independent replicates by LIVE/DEAD red dead cell viability staining and flow cytometry. (G) Geometric mean fluorescence intensity in HIV GFP+ cells following drug treatments are shown. (H) Nucleofection efficiency of siRNA knockdown in Fig 2D was determined using mScarlet reporter plasmid and flow cytometry, average of duplicate reactions was used for normalization. (I) Three replicate treatments of OM-10.1 and J-Lat cells were quantified for two housekeeping genes, GAPDH and 18S RNA, to confirm the stability of GAPDH across treatments. GAPDH CT values (delta CT [GAPDH-18S]) were compared between vehicle control and β-catenin inhibitor treated J-Lat and OM10.1 cells. Significance was determined using paired t-tests for all panels, * p<0.05, ** p<0.01, *** p<0.001. (TIF) [file ppat.1010354.s001.tif]

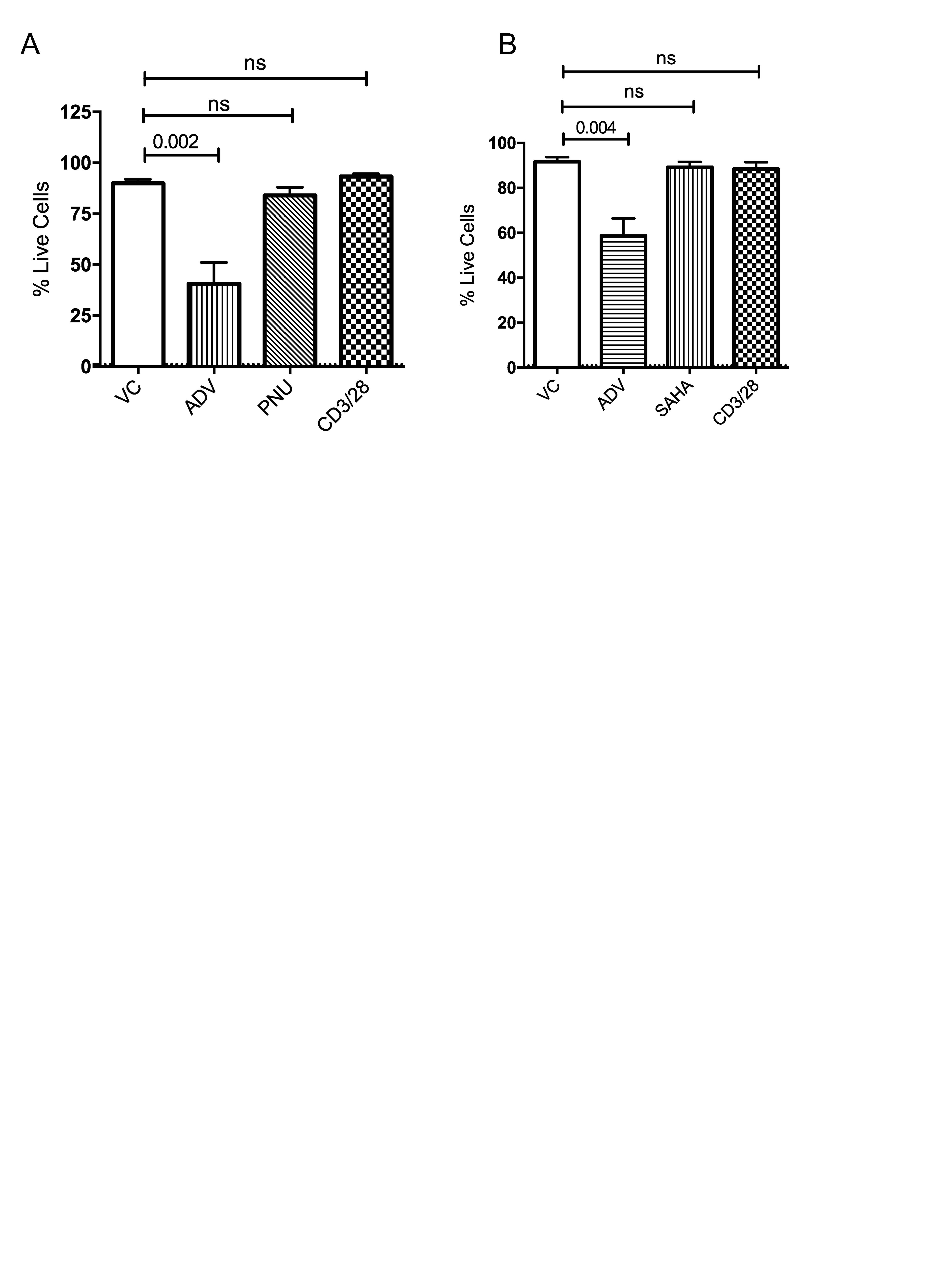

Supplement: S2 Fig — Toxicity of the drug treatments in NL4-3 (A) and REJO (B) infected cells were evaluated in each donor by LIVE/DEAD aqua dead cell viability staining and flow cytometry. Significance was determined using paired t-tests for all panels. (TIF) [file ppat.1010354.s002.tif]

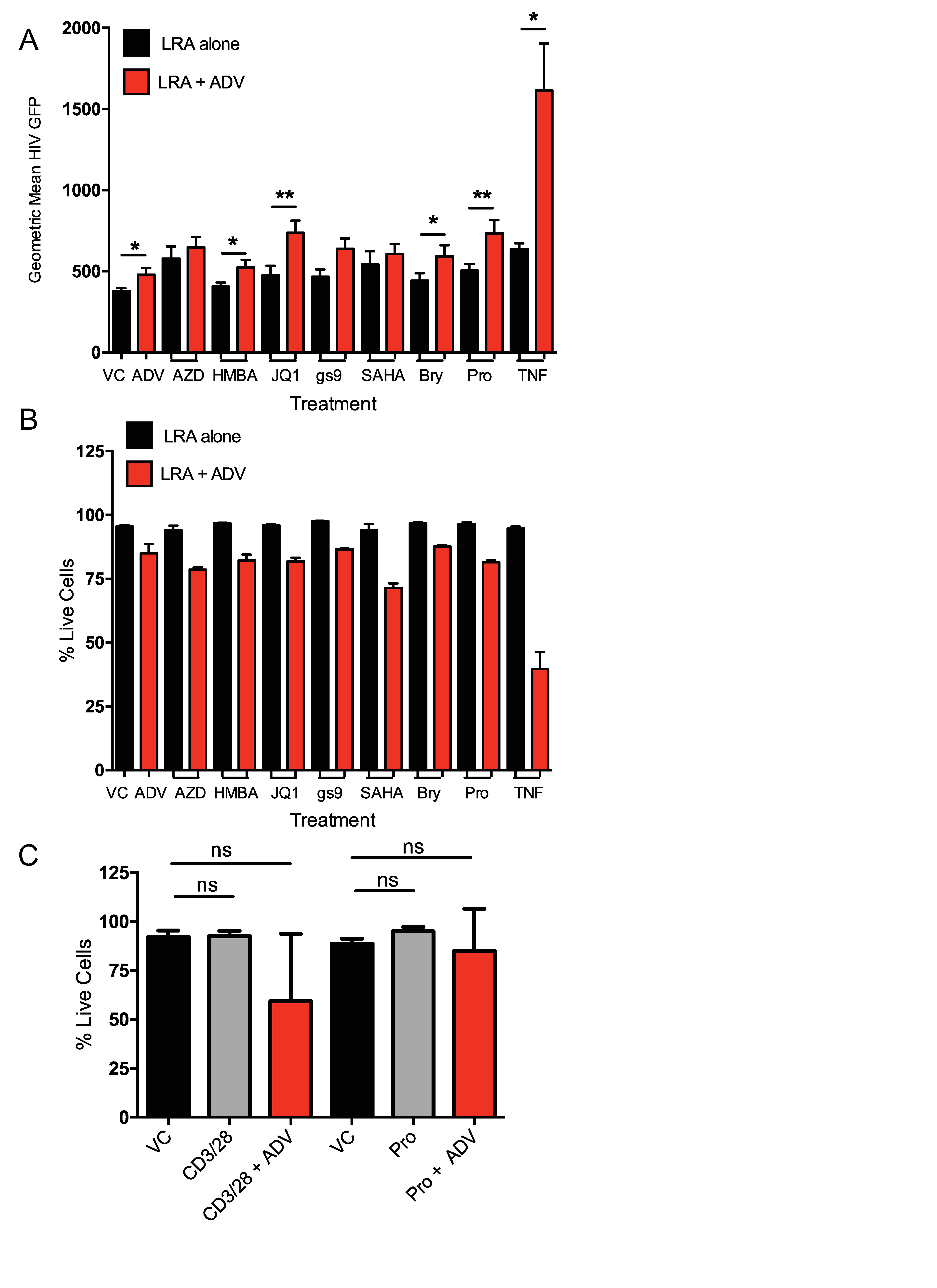

Supplement: S3 Fig — (A) Geometric mean fluorescence intensity for HIV GFP, corresponding to experiment and GFP percentages shown in Fig 4C. Significance was determined using paired t-tests, * p<0.05, ** p<0.01. (B) Toxicity of the drug treatments were evaluated in two independent replicates by LIVE/DEAD red dead cell viability staining and flow cytometry. (C) Toxicity of the drug treatments in Fig 4E were evaluated in each donor by LIVE/DEAD aqua dead cell viability staining and flow cytometry and tested for significance using paired t-tests. (TIF) [file ppat.1010354.s003.tif]

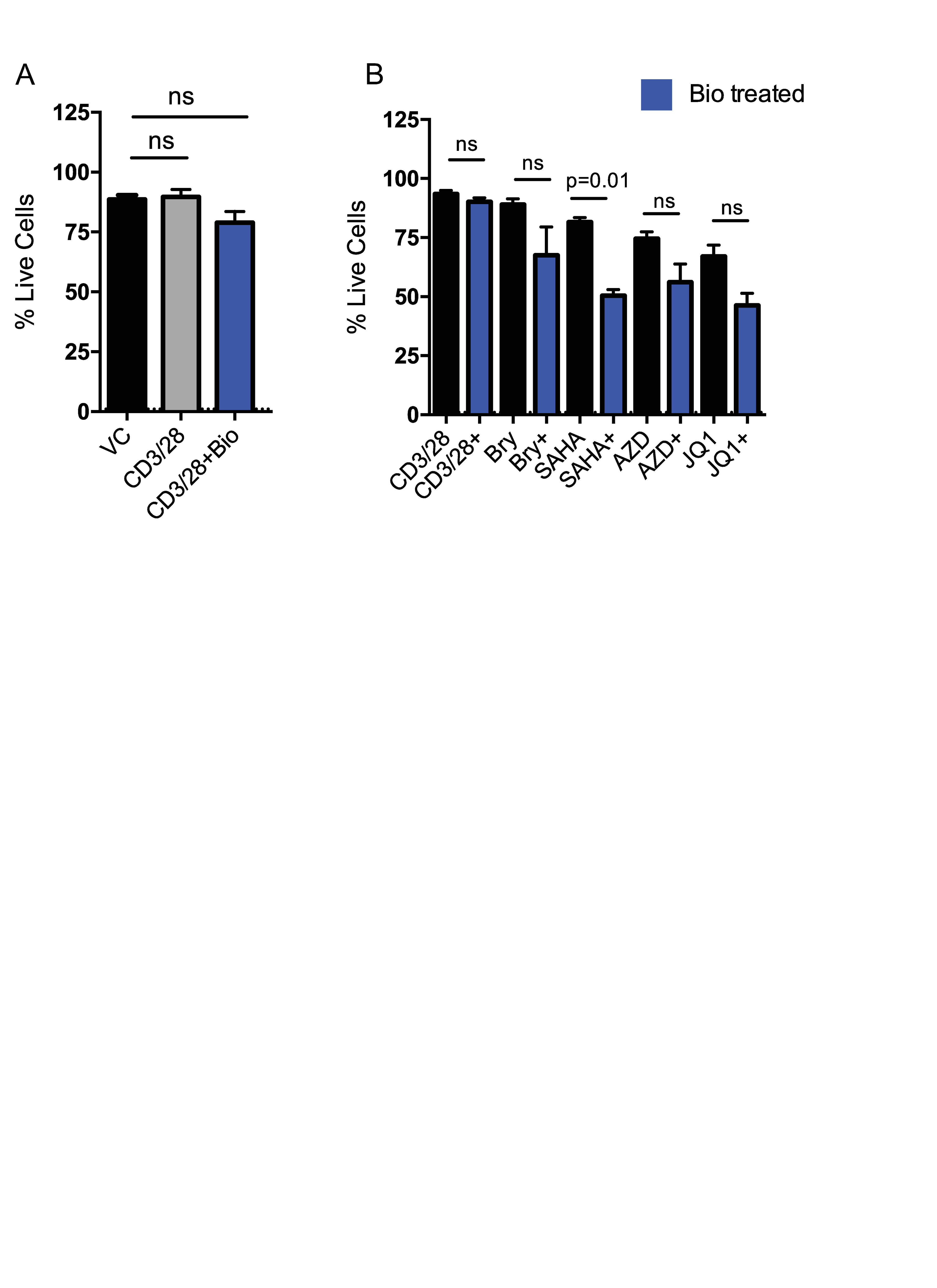

Supplement: S4 Fig — (A-B) Toxicity of the drug treatments in Fig 5 were evaluated in each donor by LIVE/DEAD aqua dead cell viability staining and flow cytometry. Significance was determined using paired t-tests for all panels, * p<0.05. (TIF) [file ppat.1010354.s004.tif]
